# Renal Response to Levosimendan in Advanced Chronic Heart Failure Patients Listed for Heart Transplantation Predicts Early Postoperative Renal Function Course

**DOI:** 10.3390/jcdd12090357

**Published:** 2025-09-16

**Authors:** Gregor Zemljic, Gregor Poglajen, Sabina Frljak, Andraz Cerar, Renata Okrajsek, Miran Sebestjen, Ivan Knezevic, Bojan Vrtovec

**Affiliations:** 1 Advanced Heart Failure and Transplantation Center, Department of Cardiology, University Medical Center Ljubljana, 1000 Ljubljana, Slovenia; gregor.poglajen@kclj.si (G.P.); sabina.frljak@kclj.si (S.F.); andraz.cerar@kclj.si (A.C.); renata.okrajsek@kclj.si (R.O.); miran.sebestjen@kclj.si (M.S.); ivan.knezevic@kclj.si (I.K.); brms.slo@gmail.com (B.V.); 2 Department of Internal Medicine, Faculty of Medicine, University of Ljubljana, 1000 Ljubljana, Slovenia

**Keywords:** heart transplantation, renal function, levosimendan

## Abstract

Background: Beyond its established inotropic effects, levosimendan has been reported to enhance renal function in patients with chronic heart failure. In this study, we investigated whether changes in renal function following levosimendan administration in patients listed for heart transplantation were associated with early post-transplant renal outcomes. Methods: We retrospectively analyzed data from 99 patients with advanced heart failure and renal insufficiency (eGFR < 90 mL/min/1.73 m^2^) who were listed for heart transplantation and received levosimendan therapy within 1 to 6 months prior to transplantation. Renal function was assessed immediately before and 24 h after levosimendan administration. A favorable renal response was defined as any increase in eGFR at 24 h. Post-transplant renal function was evaluated on postoperative days 1 and 7 using standard renal function parameters. Results: Favorable renal response to levosimendan prior to heart transplantation was present in 73 of 99 patients (74%, Group A), and 26 patients (26%) displayed no increase in eGFR (Group B). In the first week after heart transplantation, we found a significant improvement in renal function in Group A (ΔeGFR: +14 ± 3 mL/min/1.73 m^2^, *p* < 0.001), and worsening of renal function in Group B (ΔeGFR: −4 ± 3 mL/min/1.73 m^2^, *p* < 0.01). Favorable response to levosimendan prior to heart transplantation was an independent correlate of improved renal function after heart transplantation (*p* = 0.01). Conclusion: In patients awaiting heart transplantation, improvement in renal function after levosimendan therapy was associated with better early post-transplant renal outcomes. Levosimendan response may thus help identify reversible renal dysfunction and serve as a simple tool for transplant evaluation.

## 1. Introduction

In heart transplant recipients, renal dysfunction is a major determinant of both short-term and long-term outcomes, significantly increasing morbidity and mortality of these patients. It is associated with adverse events during the early post-transplant hospitalization period [1] and continues to impact survival during long-term follow-up [2]. Worsening renal function (WFR) is a common complication after heart transplantation [2], frequently progressing to end-stage renal failure requiring long-term renal-replacement therapy [3], which markedly elevates the risk of death in these patients [4,5,6].

Various risk factors are thought to be associated with worsening renal function after heart transplantation, including the use of calcineurin inhibitors, advanced recipient’s age, female sex, diabetes and hypertension [2,7]. Despite the multifactorial background of post-transplant worsening renal function, impaired renal function in advanced heart failure patients prior to heart transplantation has been identified as one of the principal predictors of post-transplant renal failure. A decline in pre-transplant estimated glomerular filtration rate (eGFR) by 10 mL/min/1.73 m^2^ has been associated with a 9% increased risk of developing chronic renal failure after heart transplantation. Moreover, greater reductions in pre-transplant GFR correlate with a progressively higher risk of post-transplant renal dysfunction [2,8].

Beyond hemodynamic and neurohormonal mechanisms, systemic and myocardial inflammation has emerged as an important driver of heart failure progression. Inflammatory pathways contribute to adverse cardiac remodeling, renal dysfunction, and poor clinical outcomes, underscoring the interplay between immune activation and end-organ damage in advanced heart failure patients [9]. In addition, recent evidence highlights the role of electrolyte disturbances—particularly the sodium-to-chloride ratio—as prognostic markers in acute and chronic heart failure, further linking metabolic and inflammatory dysregulation with adverse renal and cardiovascular outcomes [10].

In patients with advanced chronic heart failure awaiting heart transplantation, there is paucity of data on predictors of post-transplant worsening renal function. Our group has previously demonstrated that levosimendan (LS) improves long-term renal function in advanced chronic heart failure patients [11]. Additionally, perioperative administration of LS at the time of heart transplantation was associated with a lower incidence of acute kidney injury during the first post-transplant week [12].

Based on these findings, the present study aimed to investigate whether LS infusion could serve as a functional test of renal reversibility prior to heart transplantation, potentially improving and guiding heart transplant candidacy decisions.

## 2. Materials and Methods

### 2.1. Patient Population

We conducted a retrospective observational case series analysis of LS therapy-associated changes in renal function in 114 consecutive chronic heart failure patients with concomitant renal insufficiency (defined as eGRF < 90 mL/min/1.73 m^2^) who underwent heart transplantation at our center. All adult (>18 year) patients who received LS therapy within 1 to 6 months prior to heart transplantation were considered for the analysis. We excluded patients listed for multi-organ transplantation or re-transplantation, patients with short-term (ECMO) or long-term (left ventricular assist device—LVAD, total artificial heart—TAH) mechanical circulatory support prior to heart transplantation, patients with advanced chronic kidney disease (defined as eGRF < 30 mL/min/1.73 m^2^ or chronic renal replacement therapy prior to heart transplantation) and patients who did not complete follow-up evaluation 1 week after heart transplantation. Informed consent was obtained from all subjects alive at the time of data collection, while consent was waived for deceased patients. This study was conducted in accordance with the Declaration of Helsinki and approved by the National Medical Ethics Committee of the Republic of Slovenia (decision letter 0120-146/2018/16).

### 2.2. Study Design

**Figure 1:**
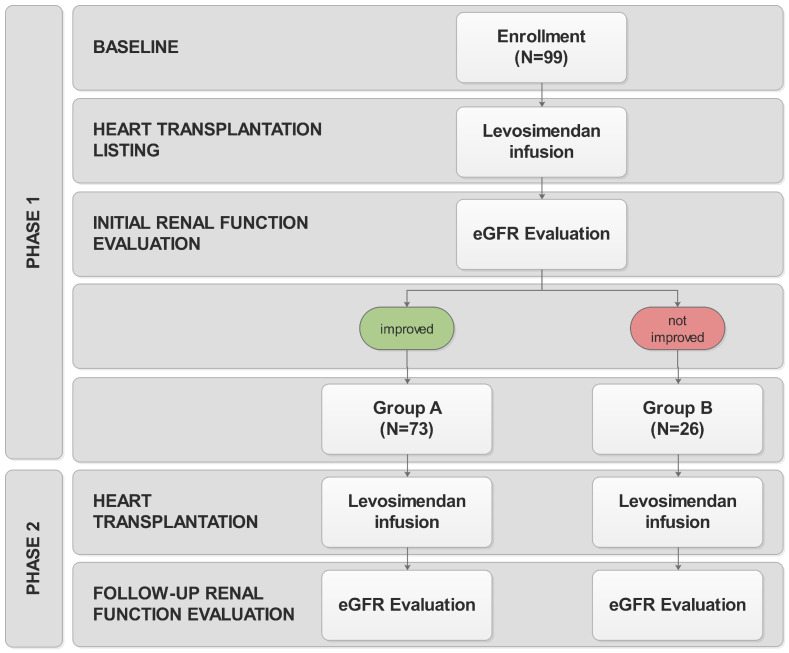
Study design flowchart. In phase 1, prior to heart transplantation, the impact of levosimendan therapy on renal function was assessed. At the time of transplantation, all patients received levosimendan. In phase 2, renal function tests were analyzed again on day 1 and 7 after heart transplantation.

Study design is outlined in Figure 1. In Phase 1, we reviewed pre-transplant parameters of renal function in advanced chronic heart failure patients on the day of LS therapy and 24 h thereafter. In all patients, LS infusion was applied at a rate of 0.1 mcg/kg/min without a bolus and the infusion of LS was continued for 24-h period. Favorable response to LS therapy was defined as any measurable increase in eGFR between baseline and 24 h after LS infusion. eGFR was estimated using the Modification of Diet in Renal Disease (MDRD) formula [13]. Patients with a favorable renal response to LS were classified as Group A, while Group B included those without improvement in renal function following LS therapy.

In Phase 2, we reviewed parameters of renal function in the same patient cohort after heart transplantation. All patients received LS infusion at the time of heart transplantation at a rate of 0.1 mcg/kg/min without bolus and the infusion of LS was continued for 24-h period. We analyzed the parameters of renal function at days 1 and 7 after heart transplantation. We analyzed the parameters of renal function at days 1 and 7 after heart transplantation. Favorable response to LS therapy was defined as any measurable increase in eGFR between days 1 and 7 post heart transplantation and 20% increase in eGFR was considered clinically significant.

### 2.3. Immunosuppression Therapy

The Immunosuppression therapy protocol consisted of basiliximab induction on day 1 and 4, methylprednisolone 1 g i.v. intraoperatively, rapidly tapered to 4 mg/day within 2 weeks post-transplant, mycophenolate mofetil at a dosage of 1000 mg b.i.d., and CNI (tacrolimus). In patients with postoperative renal dysfunction, CNI introduction was delayed, until serum creatinine had decreased to <150 μmol/L. The target tacrolimus levels were 8–10 ng/mL.

### 2.4. Statistical Analysis

Continuous variables are presented as mean ± SD, and categorical variables are expressed as number and percentage. Continuous variables were explored for normal distribution with the Shapiro–Wilk test. Differences within the groups were analyzed using a paired *t*-test for continuous variables with correction for unequal variance when appropriate, and with the chi-square or Fisher exact test when appropriate. Differences between the groups were analyzed with one-way analysis of variance (ANOVA). Repeated measurements ANOVA test was used to compare clinical results between the groups. A multivariate analysis was performed to determine the independent correlates. Statistical significance was assumed for *p* values of < 0.05. All statistical analyses were performed with SPSS software (version 22.0) (IBM, Armonk, NY, USA). More detailed information can be seen in Supplementary Materials.

## 3. Results

### 3.1. Patient Population

Of 114 reviewed patients 99 patients met inclusion criteria and were enrolled in the final analysis. A total of 15 patients were excluded from the study for the following reasons: 5 patients died waiting for heart transplantation and 3 died at less than 1-week post-transplant, 3 patients underwent mechanical ventricular assist device insertion, 2 patients were listed for heart and kidney transplantation, and 2 patients were listed for re-transplantation.

### 3.2. Renal Response to LS Therapy in Patients Listed for Heart Transplantation

**Figure 2:**
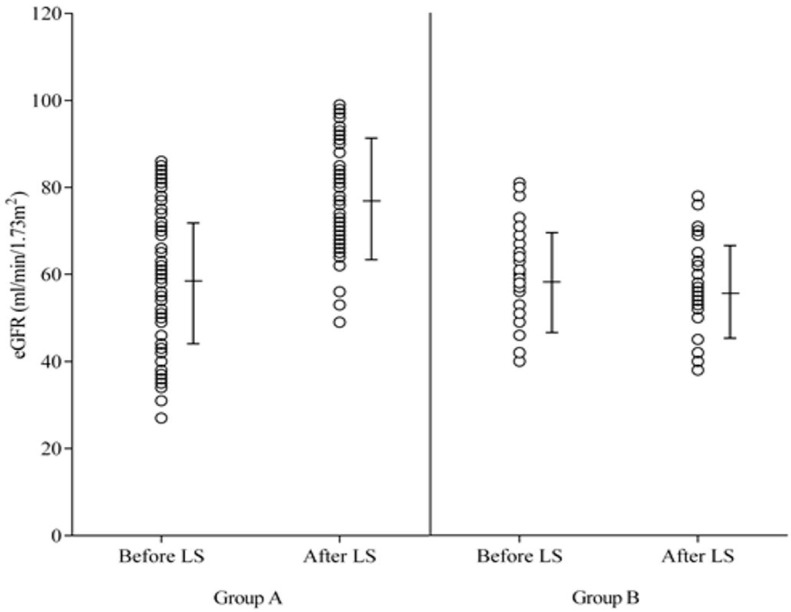
Change in the estimated glomerular filtration rate (eGFR) after levosimendan therapy in patients listed for heart transplantation. In **Group A**, renal function improved, and no increase in eGFR was found in **Group B**.

After LS therapy renal function improved in 73 of 99 patients (74%, responders, Group A), and 26 patients (26%) displayed no increase in eGFR (non-responders, Group B). Figure 2 summarizes the change in eGFR in both groups. The groups did not differ in age, gender, mean blood pressure during LS infusion, heart failure etiology, left ventricular ejection fraction, sodium, potassium, hemoglobin, leukocytes, liver function tests, or N-terminal pro-brain natriuretic peptide serum levels (Table 1).

### 3.3. Renal Response to LS Therapy in After Heart Transplantation

On day 1 after heart transplantation, renal function was comparable in both groups. We also found no differences in left ventricular ejection fraction, sodium, potassium, hemoglobin, leukocytes, liver function tests, or N-terminal pro-brain natriuretic peptide serum levels between the two groups (Table 2). Furthermore, within 1 week after heart transplantation both groups have shown comparable mean arterial blood pressure, mean tacrolimus levels, and received comparable mean prednisone dose. The rates of rejections and infections were also comparable. We also found no difference in left ventricular ejection fraction, sodium, potassium, hemoglobin, leukocytes, liver function tests, or N-terminal pro-brain natriuretic peptide serum levels between the groups (Table 3).

**Figure 3:**
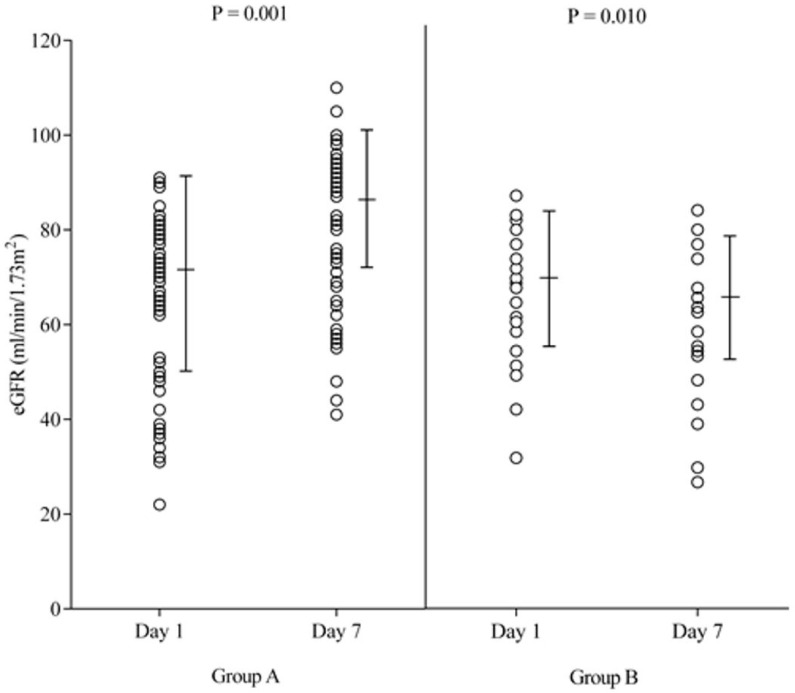
Change in the estimated glomerular filtration rate (eGFR) between days 1 and 7 after heart transplantation in both groups. We found a significant improvement in eGFR in **Group A**, and worsening of eGFR in **Group B**.

Importantly, 1 week after heart transplantation we found a significant (+20%) improvement in renal function in Group A (Δ eGFR: +14 ± 3 mL/min/1.73 m^2^, *p* < 0.001), and worsening (−6%) of renal function in Group B (Δ eGFR: −4 ± 3 mL/min/1.73 m^2^, *p* < 0.01). Figure 3 summarizes the change in eGFR in both groups. Favorable response to LS prior to transplantation was established as an independent correlate of post-transplant renal function improvement (*p* = 0.01).

## 4. Discussion

The present study evaluated whether levosimendan (LS) therapy administered prior to heart transplantation could identify advanced chronic heart failure patients with reversible renal dysfunction and predict early post-transplant renal outcomes. In a cohort of 99 patients with advanced chronic heart failure and pre-existing renal insufficiency, we found that a favorable renal response to LS therapy prior to transplantation was strongly associated with improved post-transplant renal function in the early post-transplant period. Importantly, this association remained significant despite comparable clinical, biochemical, and hemodynamic parameters between responders and non-responders. These findings support the concept that LS may serve not only as a therapeutic agent but also as a functional test for renal reversibility, potentially aiding in the risk stratification and management of transplant candidates with renal impairment.

Renal insufficiency at the time of heart transplant listing is an established risk factor for outcomes in patients after heart transplantation. Odim et al. analyzed 622 heart transplant recipients relating the risk of acute renal failure and mortality to baseline renal function. The post-transplant mortality and acute renal failure requiring hemodialysis were significantly higher in patients with pre-transplant renal insufficiency compared to patients with normal pre-transplant renal function [14]. Several other studies have also confirmed that acute renal failure requiring long-term renal replacement therapy further increases the mortality risk after heart transplantation and that the overall survival is closely related to the degree of renal impairment [15,16,17]. Nevertheless, contemporary data on testing for possible reversibility of renal impairment in advanced heart failure patients awaiting heart transplantation remain scarce. In the study by Lindelöw et al., the inotropic agent amrinone was administered to patients with advanced heart failure awaiting heart transplantation. Although renal function improved following treatment, post-transplant renal outcomes were comparable to those in patients who did not receive amrinone, and the need for hemodialysis after heart transplantation was even higher in the amrinone-treated group, likely reflecting the worse pre-transplant hemodynamic status of the latter patients [18].

Our results are consistent with the results of the previous studies that analyzed hemodynamic and clinical effects of LS therapy. In LIDO and REVIVE II studies, LS therapy improved renal function over 24-h follow-up period [19,20]. Furthermore, our group previously demonstrated a sustained improvement in renal function following LS therapy in patients with advanced chronic heart failure. At three-month follow-up, patients treated with LS showed a significant reduction in serum creatinine levels and an increase in creatinine clearance compared to untreated controls, resulting in a significant intergroup difference in serum creatinine (1.60 ± 0.26 mg/dL in the LS group vs. 1.90 ± 0.14 mg/dL in controls, *p* = 0.005) and creatinine clearance (53.6 ± 8.6 mL/min vs. 44.0 ± 3.3 mL/min, *p* = 0.005) [11]. Our results are also consistent with previous studies analyzing the effects of LS on renal function after heart transplantation. In the study of Knezevic et al., LS therapy at the time of heart transplantation was associated with significantly reduced acute kidney injury and improved renal function in the early post-transplantation period [12].

Several mechanisms have been proposed to explain the beneficial effects of LS on renal function. One hypothesis suggests that LS exerts renoprotective effects through its anti-inflammatory and anti-apoptotic properties, potentially mitigating renal ischemia–reperfusion injury [21,22,23,24]. Alternatively, the vasodilatory hypothesis is supported by the findings of Bragadottir et al., who demonstrated that LS induces vasodilation of pre-glomerular resistance vessels, thereby enhancing renal blood flow and glomerular filtration rate without compromising renal oxygenation [25,26].

Our study has several limitations. First, the study is retrospective in design with all the inherent limitations of such studies. However, the implemented study design, statistical methods used, and a large patient sample size at least partly overcome these limitations and allow confidence in the validity of the conclusions drawn. Second, we analyzed renal function with serum creatinine level and eGFR, the parameters most often used in routine clinical practice. To better define the underlying mechanisms, the measurement of other, more sensitive kidney markers such as Cystatin C would be warranted. Third, our cohort included only patients with CKD stages 2 and 3, as those with stage 1 or stages 4–5 were excluded per study criteria. Consequently, we were unable to assess potential differential responses to levosimendan across the full spectrum of renal dysfunction, which may limit the generalizability of our findings. Future studies including patients with a wider range of CKD severity will be needed to clarify whether baseline renal function stage modifies treatment response.

## 5. Conclusions

Advanced chronic heart failure patients with renal insufficiency who demonstrate improved renal function following levosimendan therapy prior to heart transplantation tend to exhibit a more favorable renal trajectory in the early post-transplant period. These findings suggest that levosimendan may serve as a useful tool to assess the reversibility of renal dysfunction at the time of transplant listing, thereby aiding in risk stratification and guiding decisions regarding combined organ transplantation.

## Figures and Tables

**Table 1:** Baseline patient characteristics.

Characteristic	All (*n* = 99)	Group A (*n* = 73)	Group B (*n* = 26)	*p* Value
Age (years)	55 ± 13	55 ± 14	54 ± 12	0.93
Male gender (%)	82 (83)	61 (84)	21 (81)	0.57
Mean arterial pressure (mmHg)	86 ± 7	85 ± 8	87 ± 6	0.76
Ischemic heart failure (%)	46 (46)	35 (48)	11 (42)	0.39
LVEF (%)	21 ± 7	22 ± 6	21 ± 7	0.62
Sodium (mmol/L)	137 ± 5	137 ± 6	138 ± 4	0.51
Potassium (mmol/L)	4.1 ± 1.1	4.1 ± 1.2	4.3 ± 1.1	0.80
eGFR (mL/min/m^2^)	56 ± 17	58 ± 17	55 ± 16	0.74
Renal function stage (%)				
Stage 1	0	0	0	/
Stage 2	48 (48)	34 (47)	14 (54)	0.67
Stage 3	51 (52)	39 (53)	12 (46)	0.56
Stage 4	0	0	0	/
Stage 5	0	0	0	/
Hemoglobin (g/L)	135 ± 17	134 ± 19	136 ± 15	0.76
Leukocyte (10^6^/mL)	7.9 ± 3.2	7.8 ± 4.2	8.4 ± 2.3	0.55
Bilirubine (μmol/L)	21 ± 16	22 ± 18	19 ± 14	0.32
AST (μkat/L)	0.73 ± 2.84	0.69 ± 3.29	0.86 ± 0.65	0.77
γGT (μkat/L)	2.51 ± 2.22	2.36 ± 1.64	2.92 ± 3.37	0.32
NT-proBNP (ng/L)	6265 ± 4638	6490 ± 4951	5632 ± 3630	0.24
Heart Failure Medical Therapy				
ARNI/ACEi/ARB (%)	96 (97%)	71 (97)	25 (96)	0.88
Beta blocker (%)	96 (97%)	70 (95)	26 (97)	0.85
MRA (%)	78 (79%)	58 (79)	20 (74)	0.74
SGLT2i (%)	50 (51%)	38 (52)	12 (46)	0.51

All values, except for p values, represent mean ± standard deviation or number of patients (percent). LVEF, left ventricular ejection fraction; AST, aspartate aminotransferase; γGT, gamma-glutamyl transpeptidase; NT-proBNP, N-terminal pro-brain natriuretic peptide; ARNI—angiotensin receptor–neprilysin inhibitor; ACEi—angiotensin-converting enzyme inhibitor; ARB—angiotensin receptor blocker; MRA—mineralocorticoid receptor antagonist.

**Table 2:** Patient characteristics on the day 1 after heart transplantation.

Characteristic	Group A (*n* = 73)	Group B (*n* = 26)	*p* Value
eGFR (mL/min/1.73 m^2^)	72 ± 30	70 ± 14	0.59
LVEF (%)	65 ± 6	63 ± 7	0.52
Sodium (mmol/L)	131 ± 9	129 ± 11	0.21
Potassium (mmol/L)	4.5 ± 0.4	4.4 ± 0.6	0.51
Hemoglobin (g/L)	122 ± 40	126 ± 42	0.73
Leukocyte (10^6^/mL)	6.4 ± 2.3	7.7 ± 1.9	0.23
Bilirubin (μmol/L)	25 ± 16	18 ± 15	0.14
AST (μkat/L)	0.87 ± 2.1	0.51 ± 0.18	0.34
gGT (μkat/L)	2.61 ± 2.03	2.07 ± 2.15	0.33
NT-proBNP (ng/L)	4794 ± 5355	4307 ± 5328	0.73

All values, except for p values, represent mean ± standard deviation or number of patients (percent). eGFR, estimated glomerular filtration rate; LVEF, left ventricular ejection fraction; AST, aspartate aminotransferase; γGT, gamma-glutamyl transpeptidase; NT-proBNP, N-terminal pro-brain natriuretic peptide.

**Table 3:** Patient characteristics within 1 week after heart transplantation.

Characteristic	Group A (*n* = 73)	Group B (*n* = 26)	*p* Value
Mean blood pressure (mmHg)	92 ± 7	90 ± 6	0.64
Mean prednisone dose (mg)	68 ± 10	72 ± 11	0.70
Mean tacrolimus level (μg/L)	9.7 ± 2.3	9.1 ± 1.9	0.78
Incidence of rejection (%)	0.04	0.03	0.70
Incidence of infection (%)	0.06	0.05	0.75
LVEF (%)	67 ± 5	66 ± 4	0.59
Sodium (mmol/L)	136 ± 8	134 ± 7	0.75
Potassium (mmol/L)	4.7 ± 0.6	4.7 ± 0.4	0.75
Hemoglobin (g/L)	132 ± 39	133 ± 32	0.92
Leukocyte (10^6^/mL)	8.5 ± 3.7	8.9 ± 2.9	0.58
Bilirubine (μmol/L)	18 ± 12	14 ± 15	0.27
AST (μkat/L)	0.44 ± 0.21	0.39 ± 0.12	0.30
gGT (μkat/L)	3.28 ± 3.65	2.46 ± 2.78	0.35
NT-proBNP (ng/L)	2834 ± 1478	3348 ± 1832	0.36

All values, except for p values, represent mean ± standard deviation or number of patients (percent). LVEF, left ventricular ejection fraction; AST, aspartate aminotransferase; γGT, gamma-glutamyl transpeptidase; NT-proBNP, N-terminal pro-brain natriuretic peptide.

## Data Availability

The raw data supporting the conclusions of this article will be made available by the authors on request.

## Supplementary Materials

The following supporting information can be downloaded at: https://www.mdpi.com/article/10.3390/jcdd12090357/s1, Table S1: The raw dataset.
